# Reconstruction of a Genome-Scale Metabolic Network for *Shewanella oneidensis* MR-1 and Analysis of its Metabolic Potential for Bioelectrochemical Systems

**DOI:** 10.3389/fbioe.2022.913077

**Published:** 2022-05-12

**Authors:** Jiahao Luo, Qianqian Yuan, Yufeng Mao, Fan Wei, Juntao Zhao, Wentong Yu, Shutian Kong, Yanmei Guo, Jingyi Cai, Xiaoping Liao, Zhiwen Wang, Hongwu Ma

**Affiliations:** ^1^ Key Laboratory of Systems Bioengineering (Ministry of Education), SynBio Research Platform, Collaborative Innovation Center of Chemical Science and Engineering (Tianjin), Frontier Science Center for Synthetic Biology (Ministry of Education), Department of Biochemical Engineering, School of Chemical Engineering and Technology, Tianjin University, Tianjin, China; ^2^ Biodesign Center, Key Laboratory of Systems Microbial Biotechnology, Tianjin Institute of Industrial Biotechnology, Chinese Academy of Sciences, Tianjin, China

**Keywords:** genome-scale metabolic model, *Shewanella oneidensis* MR-1, microbial fuel cells, microbial electrosynthesis, constraint-based flux analysis

## Abstract

Bioelectrochemical systems (BESs) based on *Shewanella oneidensis* MR-1 offer great promise for sustainable energy/chemical production, but the low rate of electron generation remains a crucial bottleneck preventing their industrial application. Here, we reconstructed a genome-scale metabolic model of MR-1 to provide a strong theoretical basis for novel BES applications. The model iLJ1162, comprising 1,162 genes, 1,818 metabolites and 2,084 reactions, accurately predicted cellular growth using a variety of substrates with 86.9% agreement with experimental results, which is significantly higher than the previously published models iMR1_799 and iSO783. The simulation of microbial fuel cells indicated that expanding the substrate spectrum of MR-1 to highly reduced feedstocks, such as glucose and glycerol, would be beneficial for electron generation. In addition, 31 metabolic engineering targets were predicted to improve electricity production, three of which have been experimentally demonstrated, while the remainder are potential targets for modification. Two potential electron transfer pathways were identified, which could be new engineering targets for increasing the electricity production capacity of MR-1. Finally, the iLJ1162 model was used to simulate the optimal biosynthetic pathways for six platform chemicals based on the MR-1 chassis in microbial electrosynthesis systems. These results offer guidance for rational design of novel BESs.

## Introduction

Growing concerns over the depletion of fossil resources and associated environmental problems have motivated the development of sustainable and renewable energy/chemical production using bioelectrochemical systems (BESs) (Apollon et al., 2022). BESs are engineered systems that utilize electrochemical interactions between microbes and electrodes, such as microbial fuel cells (MFCs) (Miyahara et al., 2013) and microbial electrosynthesis systems (MESs) (Nevin et al., 2010). In BESs, electrochemically active bacteria (EAB) play central roles in electrochemical interactions with electrodes (Logan et al., 2019). EAB can transfer electrons to or receive them from extracellularly located redox-active materials (e.g., electrodes) through so-called bi-directional extracellular electron transfer (EET) (Park et al., 2008). In MFCs, electricity is produced by EAB catalyzing the oxidation of feedstocks (e.g., organic compounds) at the electrode. MFCs offer great promise to remediate waste while producing electricity at the same time (Shi et al., 2016; Jourdin et al., 2019). In MESs, EABs accept electrons supplied by electrodes as a driving force for the synthesis of valuable chemicals (Rabaey and Rozendal, 2010; Song et al., 2019). In these processes, the rate of electron flow between electrodes and EABs is directly related to the metabolic activities of the bacteria. Consequently, it is important to study the metabolic network of an EAB and how it can be engineered to improve electricity generation in MFCs and chemical production in MESs (Hirose et al., 2019).

*Shewanella oneidensis* MR-1 has been used as a model EAB for EET research due to its remarkable respiratory versatility, which allows the bacterium to gain energy through the reduction of a variety of terminal electron acceptors, including oxygen, fumarate, nitrate, trimethylamine N-oxide (TMAO), metal oxides and electrodes (Heidelberg et al., 2002). Due to this capability, *S. oneidensis* MR-1 has been regarded as model microorganism in MFCs with many successful engineering strategies for the optimization of the EET rate from the perspectives of microbial synthetic biology (Choi et al., 2014; Sekar et al., 2016; Li et al., 2017), electrode materials (Zou et al., 2016), and bio-electrochemical reactor design (Giddings et al., 2015). In addition to transferring electrons to the anode, *S. oneidensis* MR-1 can also accept electrons from the cathode for the synthesis of chemicals such as 2,3-butanediol (Tefft and Teravest, 2019) and H_2_ (Rowe et al., 2017) in MESs. Despite these efforts, most studies to date obtained less than 40% Coulombic efficiency in MFCs (Lin et al., 2018), and the product yield of MESs was too low to make them economically competitive (Tefft and Teravest, 2019). The inefficient electron generation and transmembrane transfer are the main factors limiting the EET rate of MFCs. This might be owing to that the metabolic capacity and activity of the EABs have not been well controlled and optimized to a sufficient degree (Gong et al., 2020).

Genome-scale metabolic models (GEMs) aim to capture a systems-level representation of the entirety of metabolic functions using a stoichiometric matrix, which enables sophisticated mathematical analysis of metabolism at the level of whole-cell metabolism (Maarleveld et al., 2013). GEMs have been used to predict targets of chassis cells to enhance the production of many different chemicals (Jian et al., 2017; Gu et al., 2019; Kittikunapong et al., 2021). At present, two GEMs have been reconstructed for *S. oneidensis* MR-1. One is the iSO783 model, containing 783 genes, 870 reactions and 713 metabolites, which was published in 2010 (Pinchuk et al., 2010). The other is the updated version iMR1_799 containing 799 genes, 933 reactions and 744 metabolites reconstructed in 2014 (Ong et al., 2014). However, the number of genes contained in these two models accounted for less than 20% of annotated ORFs (18.7 and 19.0%, respectively). This resulted in low accuracy the for simulation of biological processes. For example, the iSO783 model cannot predict cell growth using threonine as carbon source, which is inconsistent with the literature (Pinchuk et al., 2010). Moreover, the periplasm compartment is not included in either of these models, yet it contains multiple respiratory chains in *S. oneidensis* MR-1. Since the publications of iSO783 and iMR1_799, numerous studies have produced new data related to gene and protein function (Hirose et al., 2019), which can be used to upgrade the gene-protein-reaction association of GEMs.

In order to facilitate internal electron generation and transfer in *S. oneidensis* MR-1, the multi-data and multi-quality-control genome-scale metabolic network model (GEM) iLJ1162 was reconstructed with significantly increased coverage of ORFs as well as accurate simulation of the accepted spectrum of substrates and electron acceptors. We employed iLJ1162 to simulate electricity generation in MFCs using different carbon sources, and identify the optimal pathways as well as overexpression targets for efficient electricity production. Finally, the production of six high-value-added chemicals in MESs using MR-1 as the chassis cell was simulated. These results offer new possibilities for the rational design of BESs.

## Materials and Methods

### Reconstruction of the Genome-Scale Metabolic Network

The genome of *S. oneidensis* MR-1 was obtained from GeneBank (GCA_000146165.2) and annotated using RAST, version 2.0 (Overbeek et al., 2005). The draft model was constructed using ModelSEED, version 2.0 (Overbeek et al., 2014). Annotation information from KEGG (Kanehisa et al., 2016), UniProt (Consortium, 2007), DeepEC (Ryu et al., 2019) and iMR1_799 was used to extended the model. For genes that were not included in the draft model but were annotated with EC numbers in KEGG, UniProt, DeepEC, or iMR1_799, reaction information used to supplement GPRs was extracted from the ModelSEED reaction database according to the corresponding EC numbers.

### Biomass Synthesis Curation

The biomass composition was adapted from the experimental data of *S. oneidensis* MR-1, which is consistent with that used in iSO783 (Pinchuk et al., 2010). To simulate biomass synthesis, the cell growth and non-growth associated ATP maintenance values (GAM and NGAM, respectively) were assumed to be identical to those used in iSO783. Gapfilling was carried out under the conditions of an l-lactate uptake rate of 4.06 mmol/gDW/h, while exchange reactions of other basic nutrient components (including H_2_O, O_2_, NH_3_/NH_4_^+^, sulfite, phosphate and metal ions) were not restricted. The gapfilling algorithm developed based on weight-added pFBA was as follows:

$$\mathrm{Min}\sum \left(abs\left(v_{i}\right)+abs\left(y_{j}\right)\right)$$ (1)

W⋅S⋅v+U⋅y=0 (2)

$$0<v_{t\arg et}\leq 10$$ (3)

$$v_{lb,\;i}\leq v_{i}\leq v_{ub,\;i}$$ (4)

$$y_{lb,\;j}\leq y_{j}\leq y_{ub,\;j}$$ (5)

where S is the stoichiometric matrix representing the model to be gap-filled, v is the vector of fluxes through reactions in S , U is the stoichiometric matrix representing the universal ModelSEED database from which reactions are activated to fill gaps, y is the vector of fluxes through reactions in U , $v_{target}$ is the flux of demand reaction for which a precursor could not be synthesized, $v_{lb,\;i}$ and $v_{ub,\;i}$ are respectively the lower and upper bounds of fluxes through reactions in the original model, W is the weight for reactions in the original model (set to 1,000), while $y_{lb,\;j}$ and $y_{ub,\;j}$ are respectively the lower and upper bounds of fluxes through reactions in ModelSEED.

The gap filling process consists of three steps. Firstly, identifying biomass compositions that cannot be synthesized. The demand reaction of each biomass composition was added and set as the optimization target for FBA calculation. If the result is 0, it means that the biomass composition cannot be synthesized, and there are gaps in its synthesis pathway. Then, the gaps for each composition were filled using the weight-added pFBA gapfilling algorithm (Eqs 1–5). The algorithm is implemented by adding 1,000 weights to all reactions in the Model_02 or Model_03 to minimize the number of gapfilling reactions introduced from ModelSEED. Finally, all the reactions used to fill the gaps are added into the Model_02 or Model_03 to enable the synthesis of all the biomass compositions.

### Phenotype Microarray Analysis and Curation of Different Nutrient Sources

The wild-type strain *S. oneidensis* MR-1 used in this study was obtained from our laboratory. The strains were stored at −80°C and revived by growing at 30 °Con LB agar plates containing (per liter): 10 g tryptone, 5 g yeast extract, and 10 g NaCl. The Biolog phenotype microarray was used to evaluate the utilization capabilities of different carbon, nitrogen, sulfur and phosphorus sources in MR-1. The PM1, PM2A, PM3B, PM4A (A-E), PM4A (F-H) plates consist of 190 carbon sources, 95 nitrogen sources, 59 phosphorus sources and 35 sulfur sources, respectively. All these plates were inoculated with cell suspensions at 100 μL/well. The plates were placed at 30°C and monitored for 72 h with readings taken at 15 min intervals. The kinetic information was recorded and quantified using OmniLog OL_FM_12 kinetic software (Biolog, United States) followed by data analysis. In the event of substrate utilization, photographic readings of color intensity resulted from dye reduction were represented in OmniLog units (OU). The substrates that were 1.5-fold higher or 0.8-fold lower than the control were selected as positive or negative results, respectively.

Gapfilling was conducted for those substrates that could be used in the Biolog experiment but not in the model. Two categories were found: 1) Substrates that lack the exchange reactions (From extracellular to intracellular). These gaps were filled manually; 2) Substrates that lack utilization pathway. These gaps were filled using the gap-filling method described above.

### Validation of the Metabolic Model

The bounds of exchange reactions were manually set to minimum medium conditions, with unrestricted H_2_O, O_2_, NH_3_/NH_4_^+^, sulfite, phosphate and metal ions. The uptake rate of the chosen carbon source was set to 4.06 mmol/gDW/h, while rates for other carbon sources were set to zero. For the simulation of terminal electron acceptors, ATPM (“rxn00062_c0”) was set as the objective function with an l-lactate uptake rate of 4.06 mmol/gDW/h. All the exchange reactions for terminal electron acceptors were turned off, but those for the chosen one ware not restricted. The metabolic model testing suite (MEMOTE) was used to evaluate the consistency and annotation of the model (Lieven et al., 2020). The validation of essential genes consists of two steps. Firstly, for each gene, the associated reactions are removed from the model; Then, biomass was set as objective function with an l-lactate uptake rate of 4.06 mmol/gDW/h. If the result is 0, it means that the gene is essential for growth.

### Constraints-Based Flux Analysis Algorithm

Flux balance analysis (FBA) (Orth et al., 2010) is used for optimizing a pre-defined objective function in the specified metabolic constraints. Unless stated otherwise, maximization of the biomass production rate was considered as the objective function. Parsimonious flux balance analysis (pFBA) (Lewis et al., 2010) was chosen for analyzing the product biosynthesis pathway. These methods were conducted using the COBRApy (0.18.1) (Ebrahim et al., 2013). The optimization solvers GLPK and Gurobi were used for linear and quadratic programming. Flux scanning based on the enforced objective flux (FSEOF) algorithm was used to identify the metabolic modification targets (Choi et al., 2010).

### Calculation of Coulombic Efficiency and Theoretical Maximum Yield

Coulombic efficiency (CE) is commonly used to quantify the performance of MFCs and is defined as the ratio of electrons actually transferred to the anode to that theoretically present in the starting carbon source (Mao and Verwoerd, 2014). For electrons exported to the anode, four reactions were added into the model to represent the electron production process. The “Electrode_Demand” reaction was set as objective to represent the generation of all electrons. Thus, CE could be represented by:

$$\mathrm{CE}\%=\frac{C_{output}}{C_{substrate}}\times 100\%=\frac{F\left(mmol/gDW/h\right)\times 2\times 100}{substrate\;uptake\;rate\;\left(mmol/gDW/h\right)\times N}\times \%$$ (6)

where F is the flux of ‘Electrode_Demand’, N is the theoretical maximum number of electrons donated by the carbon source. It can be calculated from the reduction degree of the substrate (Dugar and Stephanopoulos, 2011). For example, N for lactate is C_3_H_6_O_3_ = 3*4 + 6*1 + 3*(-2) = 12.

The theoretical maximum yield was calculated according to the ratio of reduction degrees of substrate and product.

$$\mathrm{Theoretical maximum yield}\left(\mathrm{mol/mol}\right)=\frac{C_{substrate}}{C_{product}}=\frac{N}{M}$$ (7)

where N is the reduction degree per mole of substrate, M is the reduction degree per mole of product.

## Results

### Reconstruction of a Genome-Scale Metabolic Network of *S. oneidensis* MR-1

**FIGURE 1:**
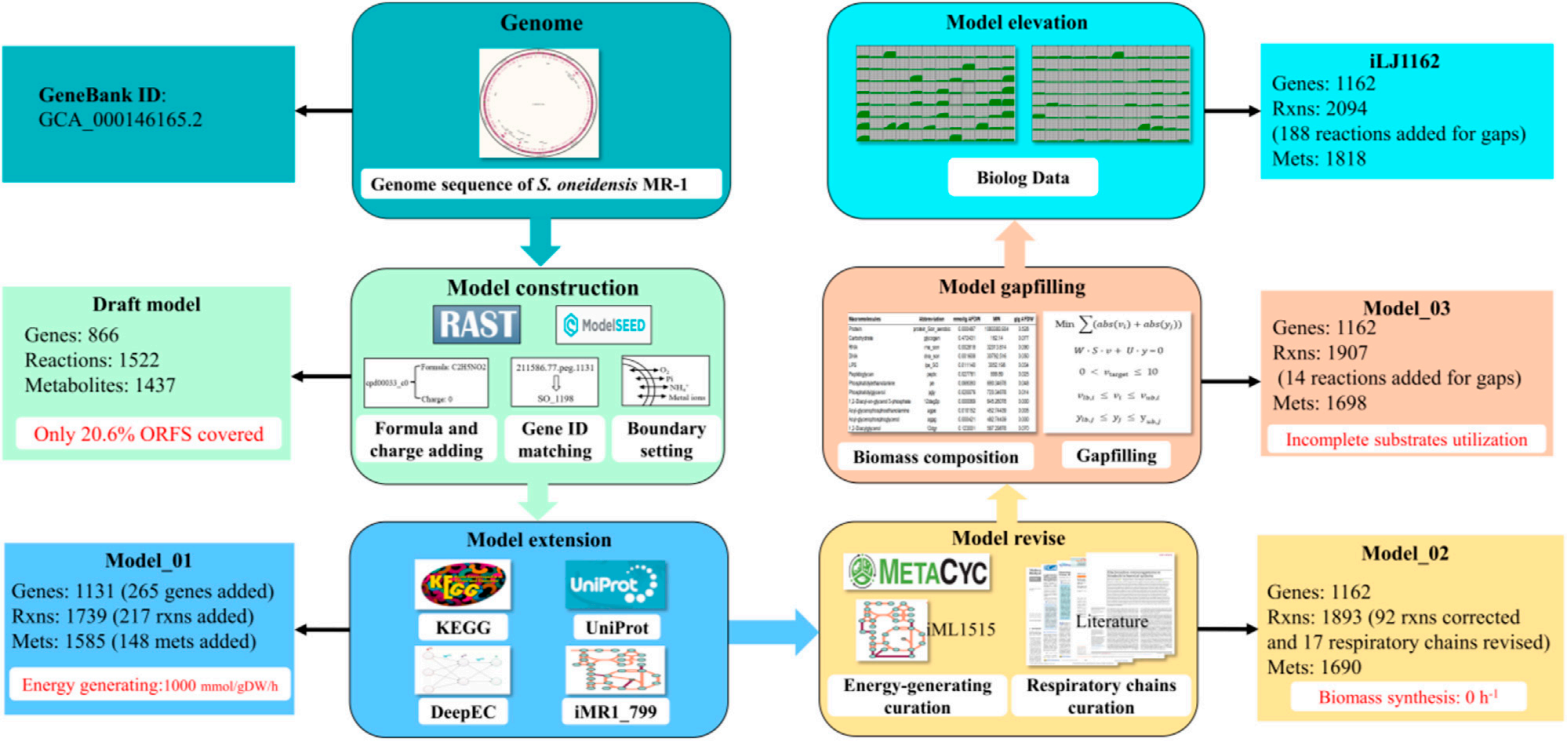
Workflow of the genome-scale metabolic network reconstruction process.

To reconstruct the genome-scale metabolic network of *S. oneidensis* MR-1, the genome of MR-1 was annotated by RAST, then uploaded into ModelSEED to generate a draft metabolic model. Charges and formulas of metabolites provided by ModelSEED were added into the draft model. The temporary gene IDs (e.g., 211586.77. peg.1131) in the draft model were transformed into specific gene IDs of *S. oneidensis* MR-1 (e.g., SO_1198) by local Blastp which was utilized for homology matching under the criteria: e-value < 1E-30; identity = 100%. The results of Blastp are listed in Supplementary Datasheet S1 (Supplementary Table S1). Furthermore, all the exchange reaction in the draft model was not constraint. Thus, the bounds of exchange reactions were manually set to minimum medium conditions, while l-lactate (4.06 mmol/gDW/h) was used as sole carbon and energy source. The draft model contained 866 genes, 1,436 metabolites and 1,524 reactions (Figure 1), which was more than the published models iSO783 and iMR1_799. For instance, dihydrofolate reductase was annotated with SO_3646 and SO_0772 in the draft model, but it was only annotated with SO_3646 in iMR1_799.

However, the draft model could only cover 20.6% (866/4196) of the annotated ORFs. Some genes, which have been reported/annotated with clear metabolic functions, were not annotated by RAST. For example, SO_4706 was annotated as malate synthase (EC: 2.3.3.9) in KEGG, but it was missed in the draft model. In order to compensate for these genes and their corresponding reactions, manual extension of the draft model was performed based on the public biochemical databases KEGG and UniProt, in combination with the published model iMR1_799. Moreover, further protein annotation from DeepEC (Ryu et al., 2019), which is a deep learning-based computational tool for prediction of EC numbers, was also used to extend the draft model. A total of 265 genes, 217 reactions and 148 metabolites were extracted to extend the draft model and generate the Model_01. All these genes and reactions can be tracked in Supplementary Datasheet S2 (Supplementary Table S2).

### Revision and Gapfilling of Genome-Scale Metabolic Network Model

**FIGURE 2:**
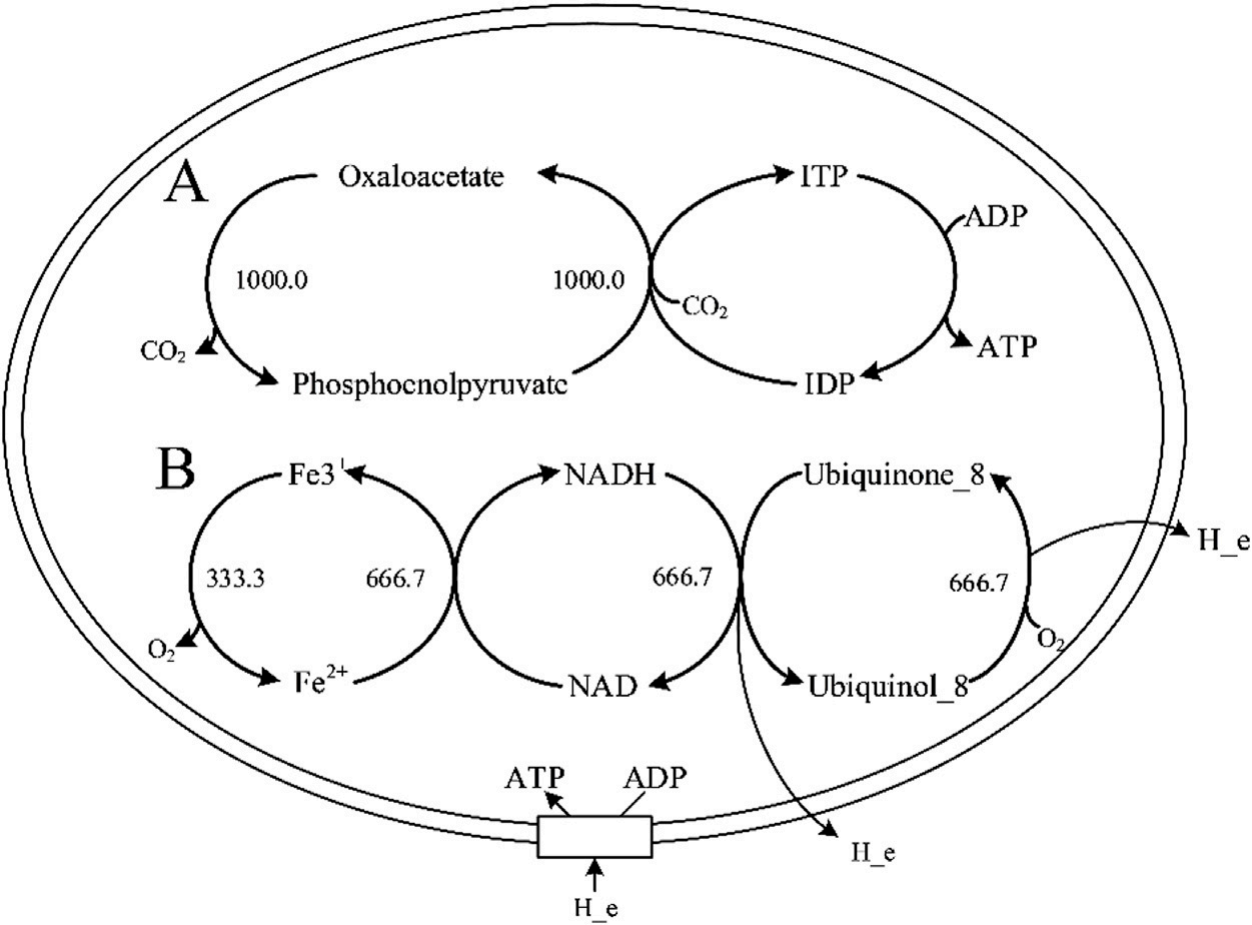
Two types of energy-generation cycles in Model_01. **(A)** represents the infinite generation of ATP in the energy-generation cycle. **(B)** represents the infinite generation of proton driving force in the energy-generation cycle. Abbreviation: ITP, inosine 5′-triphosphate; IDP, inosine 5′-diphosphate.

As shown in Figure 1, the ATP production rate (up to the upper bound of the boundary 1,000) simulated by Model_01 were incorrect. Therefore, Model_01 had to be revised. Analysis of the ATP synthesis pathway revealed that there was infinite energy-generation caused by incorrect reaction directions in the Model_01. The wrong direction of some reactions led to the infinite generation of ATP or proton driving force, resulting in energy-generating cycles (Figure 2). Therefore, the MetaCyc database (Caspi et al., 2019) and iML1515 model (Monk et al., 2017) were used as references to correct the reaction directions in Model_01. The directions of 92 reactions were modified, and energy-generating cycles were avoided (Supplementary Datasheet S1, Supplementary Table S3).

After these modifications, the optimal generation rate of ATP was changed from 1,000 to 31.3 mmol/gDW/h. The P/O of the aerobic respiratory chain in Model_01 was 1.5, which is lower than the 2.5 from iSO783 based on literature (Toledo-Cuevas et al., 1998). Thus, all aerobic respiration chains were curated based on iSO783. Consequently, a 54.5 mmol/gDW/h ATP generation rate was obtained. Furthermore, six anaerobic respiratory chains were modified and 11 anaerobic respiratory chains were added (Supplementary Datasheet S1, Supplementary Table S3). After these corrections, Model_02 was obtained (Figure 1). A total of 16 terminal electron acceptors of *S. oneidensis* MR-1, with known electron transport pathways confirmed by experimental data, were chosen for *in silico* simulations (Table 1). If the flux of energy generation is greater than 0, the terminal electron acceptor was regarded as a positive result *in silico*. The agreement of growth between the simulated results of iLJ1162 and *in vivo* results achieved 100% (16/16) of all tested electron acceptors. Nevertheless, 62.5% (10/16) and 56.25% (9/16) concordance was obtained by iMR1_799 and iSO783, respectively. This can be explained by the key reactions missing in these models. For instance, the electron transfer reaction for the conversion of AsO^4−^ was missing in iMR1_799 and iSO783, but was successfully annotated to be associated with genes *SO_4591*, *SO_1777*, *SO_1778* and *SO_1779* in iLJ1162. Notably, when using metal ions such as Fe^3+^ and Co^3+^ as terminal electron acceptors, *S. oneidensis* MR-1 obtained the highest energy synthesis efficiency *in silico*. This may be due to the reduction of these electron acceptors to generate the proton driving force and resulting energy production.

**TABLE 1:** Simulation results of 16 terminal electron acceptors by three GEMs.

Metabolites	iSO783	iMR1_799	iLJ1162	ATPM of iLJ1162 (mmol/gDW/h)	References
Fumarate	+	+	+	11.165	Maier et al. (2003)
Nitrate	+	+	+	5.329	Gao et al. (2009)
Nitrite	+	+	+	5.583	Gao et al. (2009)
DMSO	+	+	+	11.165	Gralnick et al. (2006)
TMAO	-	+	+	15.225	Dos Santos et al. (1998)
Thiosulfate	+	+	+	16.24	Shirodkar et al. (2011)
Sulfite	+	+	+	11.165	Shirodkar et al. (2011)
Fe^3+^	+	+	+	16.24	Coursolle and Gralnick, (2010)
MnO_2_	−	−	+	11.165	Bretschger et al. (2007)
CrO_4_^2−^	−	−	+	11.165	Gao et al. (2010)
UO_2_	+	+	+	16.24	Marshall et al. (2006)
AsO_4_^−^	−	−	+	4.06	Wang et al. (2016)
Pd^2+^	−	−	+	16.24	Yang et al. (2020)
V^5+^	−	−	+	16.24	Myers et al. (2004)
Co^3+^	+	+	+	16.24	Hau et al. (2008)
Cu^2+^	−	−	+	16.24	Shen et al. (2017)

The ‘+’ and ‘−’ symbols indicate that ATPM, flux was obtained or not *in silico*, respectively.

However, the simulated growth rate of Model_02 was still zero, while that the reported experimentally measured growth rate of *S. oneidensis* MR-1 cultured in minimal medium was 0.085 h^−1^, and the corresponding l-lactate consumption rate was 4.06 mmol/gDW/h under aerobic conditions (Pinchuk et al., 2010). This can be explained by the inability of Model_02 to synthesize a number of biomass precursors, such as lipopolysaccharide and glycogen. Therefore, the weight-added pFBA algorithm was used to fill the gaps in Model_02 (Materials and Methods). A total of 14 reactions (Supplementary Datasheet S1, Supplementary Table S4) were added into Model_02 to generate Model_03. Under the same l-lactate uptake rate, the predicted cell growth rate of iSO783 was 0.108 h^−1^ while that of iMR1_799 was 0.192 h^−1^. However, Model_03 predicted a rate of 0.105 h^−1^, which was the closest to the actual *in vivo* value of 0.085 h^−1^ (Supplementary Datasheet S1, Supplementary Figure S1).

**FIGURE 3:**
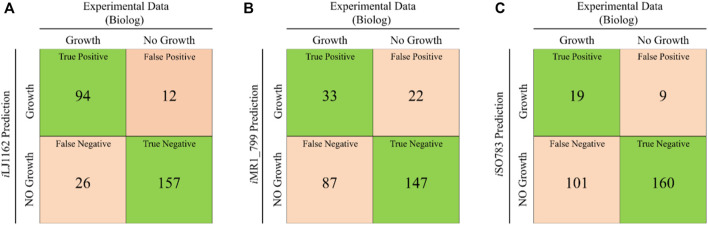
Comparison of *in silico* simulations and *in vivo* results for the utilization of various substrates. **(A)** The comparison results of iLJ1162. **(B)** The comparison results of iMR1_799. **(C)** The comparison results of iSO783. Green square represents correct predictions, and orange square represents incorrect predictions.

In order to improve the substrates utilization capacity of Model_03, 190 carbon sources, 95 nitrogen sources, 59 phosphorus sources and 35 sulfur sources were systematically investigated in Biolog phenotype microarray experiments (Supplementary Datasheet S1, Supplementary Table S5). The Biolog results showed that 27 carbon sources, 32 nitrogen sources, 47 phosphorus sources and 14 sulfur sources can be used by *S. oneidensis* MR-1. However, 54 of these could not be utilized by Model_03 due to missing transport or utilization reactions (Supplementary Datasheet S1, Supplementary Table S6). After supplementing 187 reactions obtained by the weight-added pFBA algorithm, iLJ1162 was generated (Supplementary Datasheet S2). A total of 289 substrates were used to evaluate the substrate utilization abilities in different models. 94 true positive (substrates that can be used in both the *in vivo* experiments and simulations) and 157 true negative (substrates that cannot be used in both the *in vivo* experiments and simulations) results were obtained by iLJ1162 (Figure 3). This means iLJ1162 has an agreement of 86.9% with the experimental data, which is significantly higher than the 62.3 and 61.9% obtained by iMR1_799 and iSO783, respectively (Figures 3B,C). This can be attributed to the extensively extended GPRs associated information and gapfilling curation for these substrates (Supplementary Datasheet S1, Supplementary Table S6). However, due to current limited understanding of metabolism of some substrates, there are still some inconsistencies of *in vivo* results with those obtained by iLJ1162. For instance, the utilization pathways of Tween 20, 40, and 80 as well as gelatin, are still unknown, and these cannot be simulated *in silico*.

**FIGURE 4:**
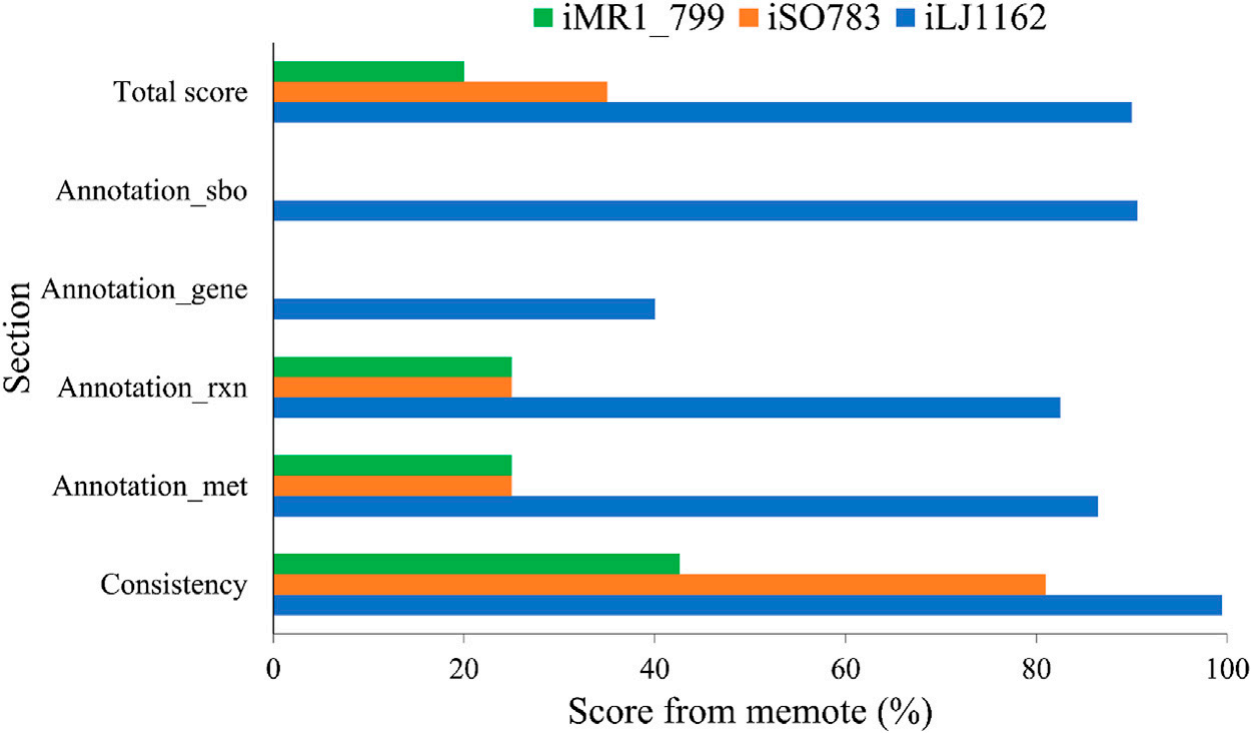
Evaluation of genome-scale metabolic models of *S. oneidensis* MR-1 using MEMOTE.

The final version of the model, iLJ1162, includes 1,162 genes, 1818 metabolites and 2084 reactions, which were divided into the three extracellular, periplasmic and cytosolic compartments. MEMOTE was used to assess the quality of iLJ1162 and the other two published GEMs of MR-1 (Supplementary Datasheet S3). In addition to the significant improvements in consistency as well as annotation of reactions and metabolites, iLJ1162 has clear annotations for genes and SBO-terms (Systems Biology Ontology) (Figure 4). These result in a clear advantage of iLJ1162 in the total score. All validation results confirmed the high reliability of iLJ1162, indicating that it can be used for further analysis.

### Analysis of Essential Genes of *S. voneidensis* MR-1

Essential genes are an important characterization of model quality (Lee et al., 2014; Nouri et al., 2020). Therefore, iLJ1162 was used to analyze the essentiality of individual genes of MR-1 under aerobic conditions (Materials and Methods). From the report by Yang et al. (2014), we collected 781 unambiguous gene inactivation results whose gene IDs could be matched to the three MR-1 models. After comparison of *in vivo* results with *in silico* simulations, iLJ1162 achieved an accuracy of (681/781) 87.2%, significantly higher than (471/781) 60.3% of iSO783 and (477/781) 61.1% of iMR1_799 (Supplementary Datasheet S4, Supplementary Table S7). This can be majorly attributed to the significantly improved ORFs coverage in iLJ1162. However, there were still some inconsistent results, including 59 false negative results (essentiality for experiment, but non-essentiality for iLJ1162) and 35 false positive results (essentiality for iLJ1162, but non-essentiality for experiment). For these false negative results, it is probably caused by the cellular transcriptional regulation in MR-1. For example, SO_3415 was annotated as aspartate kinase and assessed as essential gene from the *in vivo* experiments. However, in the iLJ1162, there exist other three isoenzymes (*SO_4055*, *SO_3427* and *SO_3986*), which might be strictly regulated and unexpressed in MR-1. As for false-positive results, it is likely caused by the incomplete annotation (coverage of ORFs, 27.8%) in iLJ1162. For example, *SO_4234* was annotated as 3-isopropylmalate dehydratase and participates an essential role in l-leucine synthesis in all the three models. If the results of the experiment are accurate, MR-1 may have potential isoenzymes for 3-isopropylmalate dehydratase or alternative pathways for leucine synthesis.

### Simulation of Electrons Generation Using Different Substrates

**FIGURE 5:**
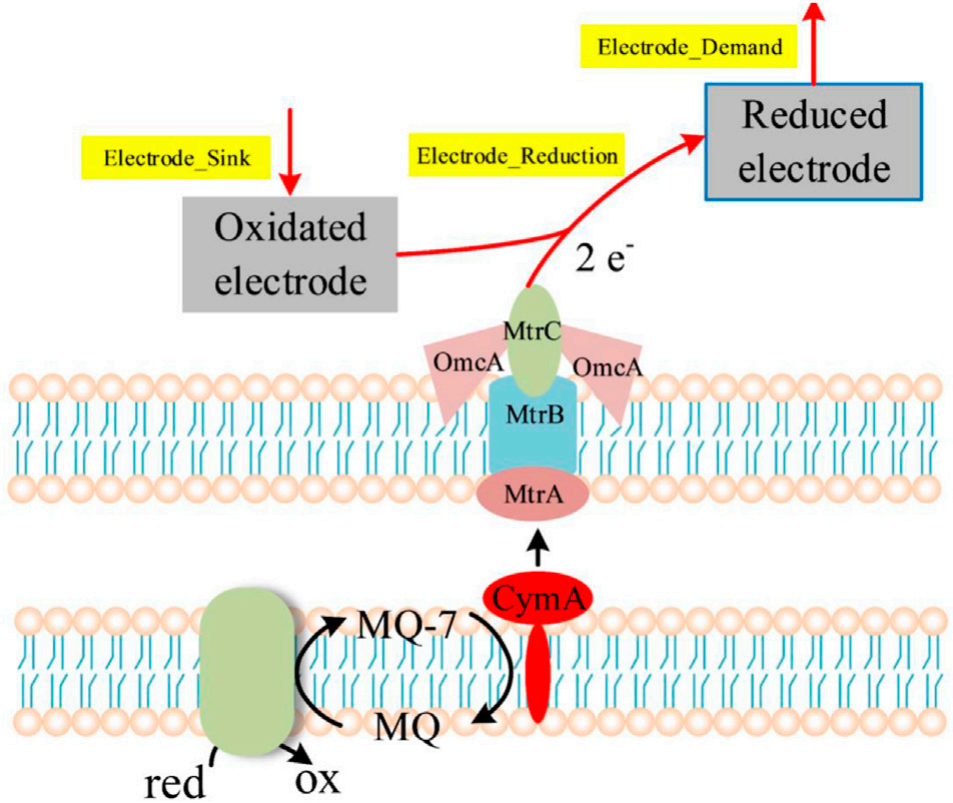
Respiratory electron transport in MFCs based on *S. oneidensis* MR-1. The arrows in the dotted box represent the reactions used for the simulation of electron generation.

In MFCs, electrons obtained through the oxidation of the substrates are transferred to electrodes via the electron transport chain (Figure 5). The electrode is actually one of terminal electron acceptors, and can be set as a pseudo-metabolite which is similar to metal ions such as Fe^3+^ (Mao and Verwoerd, 2014). Thus, four artificial reactions (Electrode_Sink, → Oxidated_Electrode; Electrode_Reduction_1, 2.0 Oxidated_Electrode + mql7 → 2.0 Reduced_Electrode + Menaquinone_7 + 2.0 H_plus__e0; Electrode_Reduction_2, 2.0 Oxidated_Electrode + methymenaquinol_7 → 2.0 H_plus__e0 + 2.0 Reduced_Electrode + methylmenaquinone_7; Electrode_Demand, Reduced Electrode →) were added into the model for simulation of electron production (Supplementary Datasheet S4, Supplementary Table S8). The “Electrode_Demand” reaction was set as the objective function to represent the generation of electrons.

To simulate the electron generation ability of MR-1 with different substrates, 15 available carbon sources experimentally validated using Biolog PM1-2 and five heterogenous carbon sources widely used as laboratory substrates were chosen for computational simulation. Taking both the biomass synthesis and electron generation into consideration, the biomass growth rate was set to 10% of the maximum theoretical value, then the maximization of electron generation was set as objective function. This is consistent with that MR-1 grows slowly in MFCs under anaerobic conditions (Logan et al., 2019).

**FIGURE 6:**
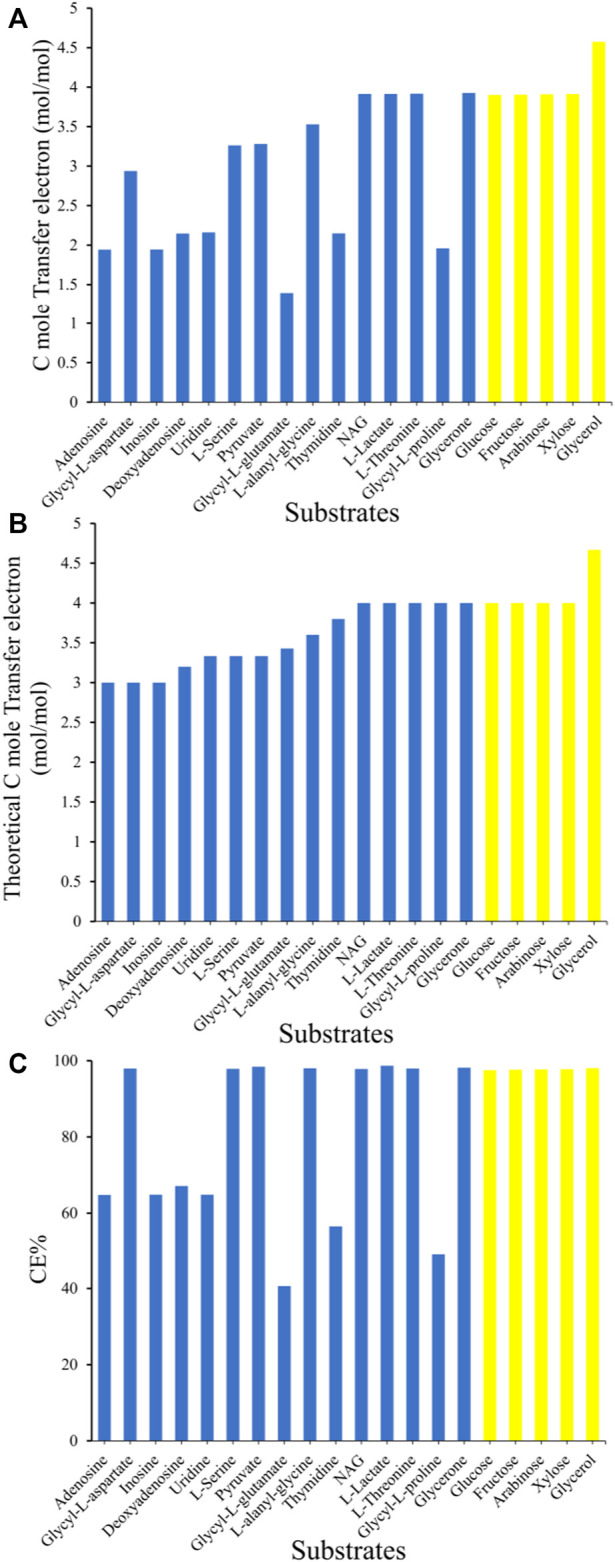
Comparison of the electron generation capability of *S. oneidensis* MR-1 with 20 different carbon sources *in silico*. **(A)** The simulated electron generation rate per C-mole substrate. **(B)** The inherent electron numbers per C-mole substrate. **(C)** The Coulombic efficiency (CE). The blue columns indicate substrates that are naturally utilized by MR-1, and the yellow columns indicate substrates that require the introduction of heterologous utilization pathways.

In general, the simulated electron generation rate per C-mole substrate (Figure 6A) was positively correlated with the inherent electron number per C-mole substrate (Figure 6B). Glycerol was predicted to be the optimal substrate for electron generation with the highest rate (4.57 mmol per C-mol glycerol), which was ∼15% higher than that of the common laboratory common substrates l-lactate or N-Acetyl-d-glucosamine (NAG). Many substrates obtained high CE values over 95%, which means that the electrons of these substrates can practically to be fully transferred to the electrode by the metabolic network of MR-1 (Figure 6C). However, some substrates such as adenosine and inosine generated low C-mole electron generation rates despite their high inherent electron numbers (Figures 6A,B), which led to low CEs values from 40 to 70%. This is attributed to the production of intermediate metabolites during the metabolism of these substrates, such as hypoxanthine production for adenosine and inosine utilization, or succinate production for the utilization of the dipeptide glycyl-l-proline. However, these intermediate metabolites cannot be further metabolized by MR-1 under anaerobic conditions, and have to be transported outside of the cell, causing electron waste.

### Simulation of Intracellular Regeneration of Reducing Equivalents

**FIGURE 7:**
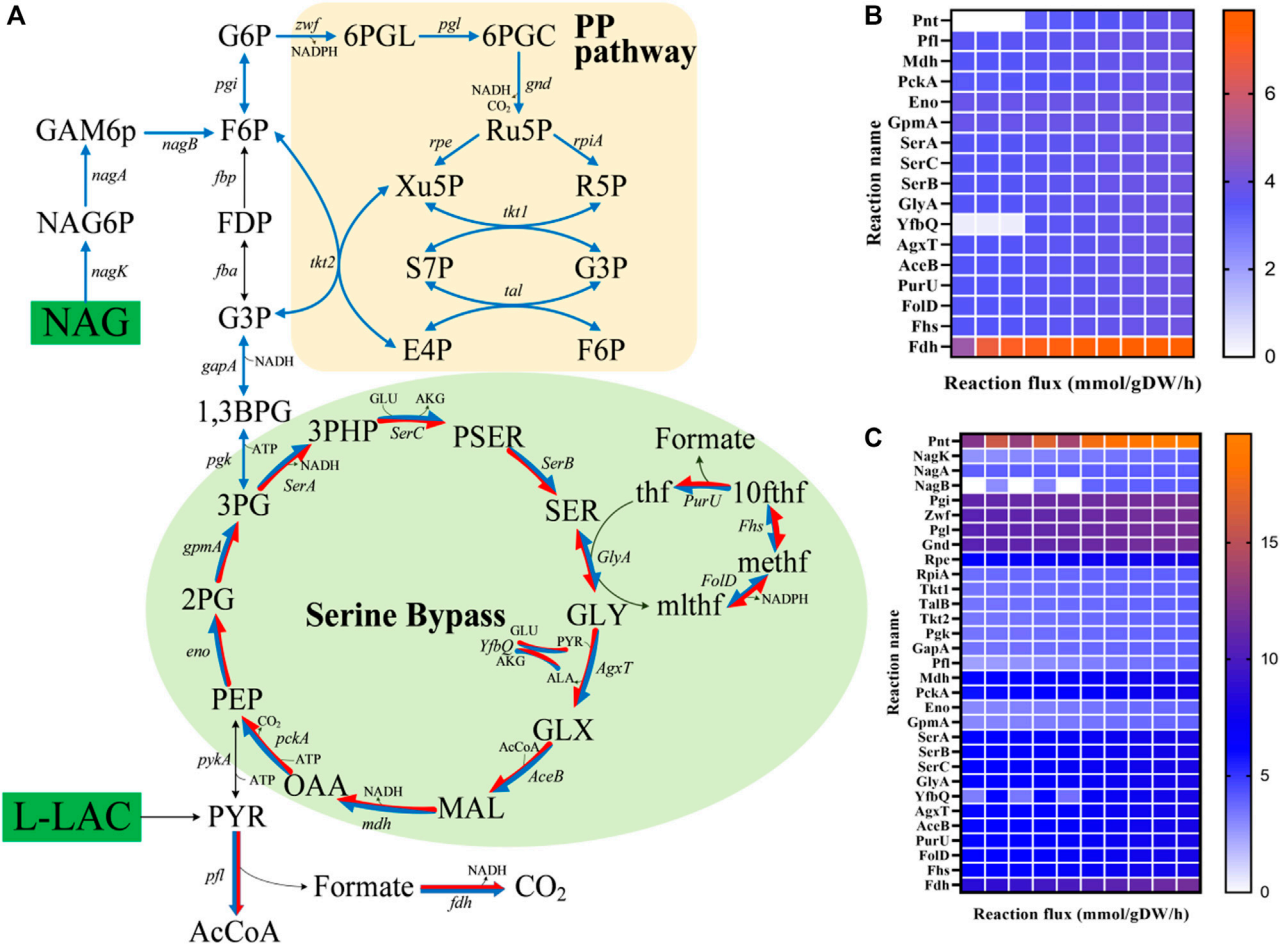
FSEOF analysis for NADH regeneration. **(A)** Distribution of targets predicted by the FSEOF algorithm. The green module represents the serine bypass, and the orange module represents the pentose phosphate pathway (PP pathway). Blue arrows represent overexpression targets when NAG was used as the substrate, and red arrows represent overexpression targets when l-lactate was used as the substrate. **(B)** Reaction flux changes of targets with an l-lactate uptake rate 4.06 mmol/gDW/h. **(C)** Reaction flux changes of targets with an NAG uptake rate of 4.06 mmol/gDW/h. Abbreviation: f6p, d-fructose 6-phosphate; fdp, d-fructose 1,6-bisphosphate; g3p, glyceraldehyde 3-phosphate; 1,3bpg, 1,3-Bisphospho-d-glycerate; 3pg, 3-phospho-d-glycerate; 2pg, 2-phospho-d-glycerate; pep, phosphoenolpyruvate; pyr, pyruvate; g6p, d-glucose 6-phosphate; 6pgl, 6-phospho-D-glucono-1,5-lactone; 6pgc, 6-phospho-d-gluconate; accoa, acetyl-CoA; akg, 2-oxoglutarate; oaa, oxaloacetate; mal, malate; glx, glyoxylate; 3php, 3-phosphonooxypyruvate; pser, phosphoserine; 10fthf, 10-formyltetrahydrofolate; methf, 5,10-methenyltetrahydrofolate; mlthf, 5,10-methylenetetrahydrofolate; Ser, l-serine; thf, 5,6,7,8-tetrahydrofolate; ru5p_D, d-ribulose 5-phosphate; xu5p_D, d-xylulose 5-phosphate; r5p, d-ribose 5-phosphate; s7p, sedoheptulose 7-phosphate; e4p, d-erythrose 4-phosphate.

The enhancement of the regeneration of reducing equivalents, mostly in the form of NADH, has been reported to be one of the most successful metabolic engineering strategies for the improvement of MFCs (Li et al., 2018). In view of the important role of NADH regeneration in MFCs, the iLJ1162 model was used to predict potential modification targets for increasing the NADH regeneration capacity of *S. oneidensis* MR-1 using the FSEOF algorithm (Choi et al., 2010). An artificial reaction (NADH_G, NADH_c → NAD^+^_c + H^+^_c) was introduced into the model and used as the optimization objective to calculate the optimal pathways for NADH regeneration from NAD^+^. In addition, since there is low or no measurable activity of the pyruvate dehydrogenase (PDH) encoded by SO_0424 and SO_0425 in *S. oneidensis* MR-1 under anaerobic conditions (Yang et al., 2006), the reactions corresponding to PDH were temporarily turned off. In the simulation, the reactions with significant flux changes from optimal biomass synthesis to optimal NADH regeneration were chosen as potential targets. Two common laboratory substrates for *Shewanella* species, l-lactate and NAG were introduced as simulated substrates (Supplementary Datasheet S4, Supplementary Table S9). In the modification strategies obtained from the FSEOF simulation with l-lactate as substrate, 17 overexpression targets were mainly distributed in the serine cycle (Figures 7A,B). Among them, NAD^+^ dependent malate dehydrogenase (Mdh), pyruvate formate-lyase (Pfl) and NAD^+^ dependent formate dehydrogenase (Fdh) were consistent with previously reported experimental results (Li et al., 2018), which means that the modification strategies predicted by iLJ1162 are meaningful. Thus, other targets may be potentially effective and worth implementing to improve electron production *in vivo*. In the case of NAG (Figures 7A,C), the simulation results revealed that 31 potential targets, mainly distributed in the PP pathway (e.g., Rpe and RpiA) and serine bypass (e.g., SerA and Mdh).

### Prediction of Potential NADH Dehydrogenases for Improving EET Rate

**FIGURE 8:**
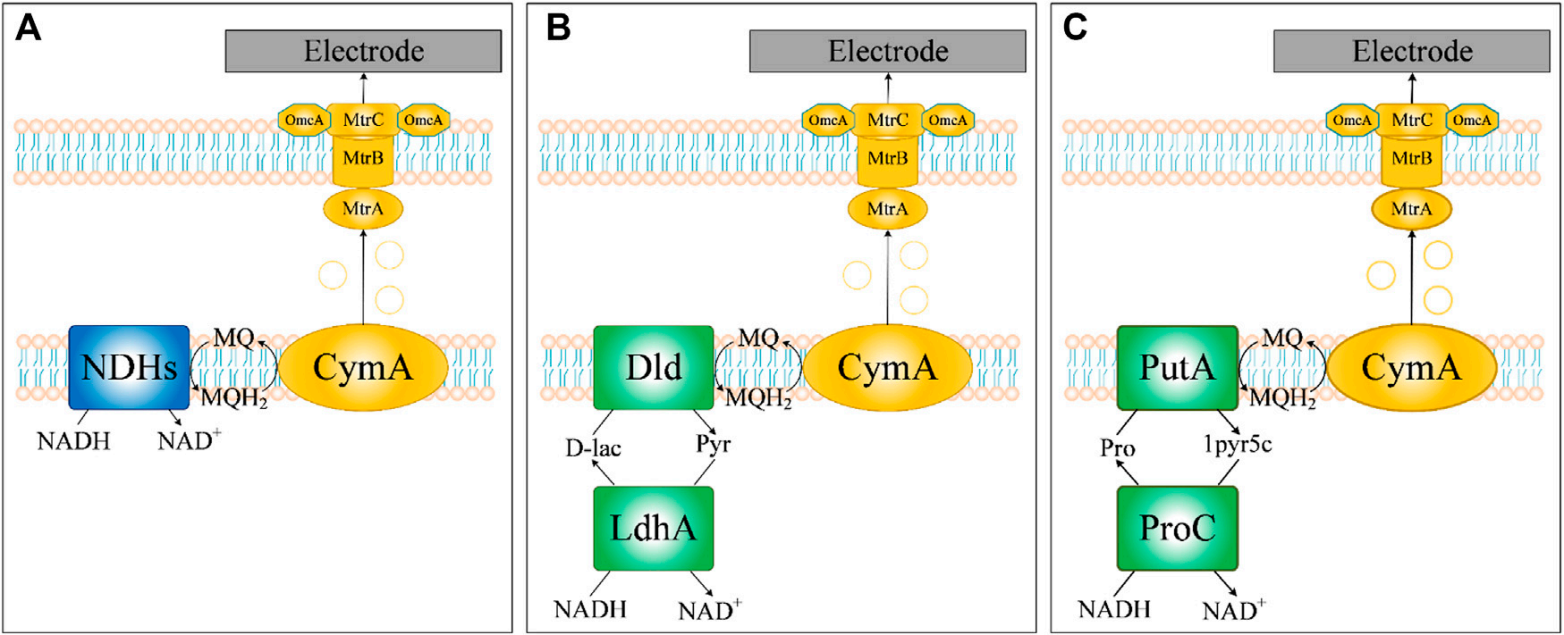
Three NADH dehydrogenases predicted by iLJ1162 for electron transfer in *S. oneidensis* MR-1. **(A)** Electron transfer based on NADH dehydrogenases. **(B)** Electron transfer based on LdhA-Dld. **(C)** Electron transfer based on ProC-PutA.

An important approach for increasing EET rates based on high intracellular regeneration of NADH is the transfer of electrons from NADH to the respiratory chain. The first step of the respiratory chain is the dehydrogenation of NADH by NADH dehydrogenases (NDHs) to release electrons, which are important for energy supply and cell growth (Figure 8A). Three types of prokaryotic NADH dehydrogenases are generally recognized in the literature (Melo et al., 2004), including those with proton-pumping activity (type I), proton/sodium-pumping activity (type II) and sodium-pumping activity (type III). Notably, the genome of *S. oneidensis* MR-1 encodes all three types of NDHs (type I: SO_1009-SO_1021, type II: SO_1103-SO_1108 and SO_0902-SO_0907, type III: SO_3517). It has been reported that electrical current production or growth rate with NAG as the substrate under anaerobic conditions would be extremely inhibited by knocking out NDHs (Kasai et al., 2019; Madsen and Teravest, 2019). However, the simulation results of iLJ1162 indicated that there was no obvious decrease of electron production or growth rate. Further analysis revealed that there are two alternative pathways with NDHs function, including 1) LdhA (pyruvate reductase encoded by SO_0968, EC: 1.1.1.28) and Dld (lactate dehydrogenase by SO_1521, EC: 1.1.2.4) (Figure 8B); 2) ProC (pyrroline-5-carboxylate reductase encoded by SO_3354, EC: 1.5.1.2) and PutA (proline dehydrogenase encoded by SO_3774, EC: 1.5.5.2) (Figure 8C). The former has been experimentally verified (Kasai et al., 2019), while the latter was uncovered by iLJ1162 for the first time. However, the two pathways failed to play a role in electricity production or cell growth, probably due to the strict transcriptional regulation and low pools of intermediate metabolites (d-lactate or l-proline) (Kasai et al., 2019; Madsen and Teravest, 2019). Thus, overexpression of NDHs and/or activation of LdhA-Dld/ProC-PutA might be an effective strategy for channeling more electrons toward MQ to improve EET rate.

### Simulation of the Synthesis of High-Value-Added Products by iLJ1162

In MES systems, electrons from the cathode are transferred to the microbial cells and used for the synthesis of value-added chemicals (Logan et al., 2019). The MES process of *S. oneidensis* MR-1 can be summarized as NADH generation by receiving extracellular electrons and protons using NAD^+^ (Yang et al., 2015; Rowe et al., 2017). Thus, the reaction of “NADH_R” (2.0 e^−^ + H_p + NAD_c → NADH_c) and ‘Sink_e^−^’ (→ e^−^) were added to represent the electron receiving process. Six high value products were chosen for simulation in MESs, including 3-hydroxypropanoate (3HP), isopropanol (2ppoh), 3-hydroxybutanoate (3HB), acetoin (Actn), succinate (Suc), and 2,3-butanediol (23btdl). All these products require no more than two heterologous reactions in MR-1. The cell growth was omitted to simplify the complexity of biosynthesis pathways for different products, which is also consistent with that EAB almost did not grow in MESs (Chen et al., 2018; Tefft and Teravest, 2019) The demand reactions of these products are set as objective function respectively.

**FIGURE 9:**
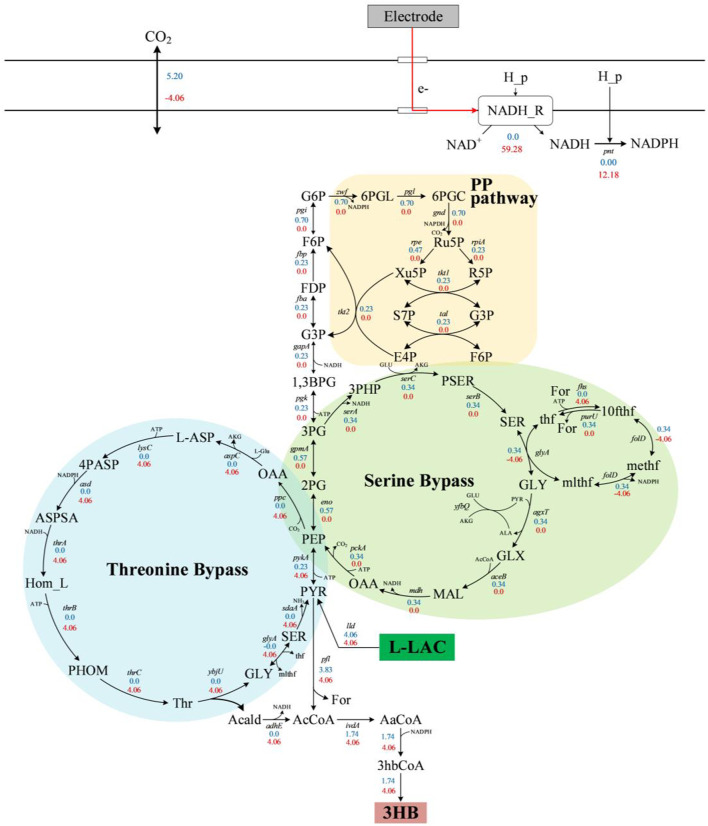
Simulation results of the flux distribution for 3HB synthesis. Blue or red fluxes represent iLJ1162 simulation results without or with inward electrons. The blue module represents the threonine bypass, the green module represents the serine bypass and orange represents the pentose phosphate pathway. Abbreviation: For, formate; L-asp, l-aspartate; 4pasp, 4-phospho-l-aspartate; aspsa, l-aspartate 4-semialdehyde; hom_L, l-homoserine; phom, O-phospho-l-homoserine; Thr, l-threonine; acald, acetaldehyde; AaCoA, acetoacetyl-CoA; 3hbCoA, 3-hydroxybutanoyl-CoA.

The simulation results of these six products in MESs using l-lactate as sole carbon source under anaerobic conditions are shown in Table 2. Without inward electron (no extra electron supply), the simulated yields were lower than their theoretical maximum yields calculated from reducing degrees. However, with inward electrons, all products achieved or exceeded the theoretical maximum yields calculated from the reduction degrees. In the simulation of MESs, CO_2_ is infinitely supplied, which is consistent with the actual MESs aerated with CO_2_ to ensure a strict anaerobic condition (Chen et al., 2018). Thus, with extra electron supply (inward electrons), it is theoretically feasible to fix environmental CO_2_, and consequently break mass balance merely based on substrate. As one of the C4 platform chemicals, 3-hydroxybutanoate (3HB) was chosen as an example for MESs simulation using iLJ1162. As shown in Figure 9, a simulated yield of 0.43 mol/mol was obtained using l-lactate as the sole electron source, which is only 64% of the theoretical maximum yield calculated from reducing degrees. This can be explained by the fact that the key intermediate metabolite formate cannot be further converted to 3HB by the metabolic network of MR-1, resulting in the loss of one third of total carbon in l-lactate. Moreover, to obtain NADPH for 3HB synthesis, a part of carbon fluxes was directed into the serine bypass and PP pathway, and oxidized to release electrons for NADPH generation.

**TABLE 2:** Simulation results for the synthesis of six products in MESs.

Products	Yield (mol/mol l-Lactate)				Inward electrons (mmol/gDW/h)
	Without inward electrons	With inward electrons	TMY	Carbon-Mole TMY	
3HP	0.518	1	1	1	21.112
2ppoh	0.484	1	0.667	0.667	118.552
3HB	0.43	1	0.667	0.5	118.552
actn	0.5	0.667	0.6	0.45	42.7654
suc	0.563	1.5	0.857	0.643	170.52
23btdl	0.417	0.667	0.545	0.409	57.923

TMY, represents theoretical maximum yield calculate from reducing degree.

With inward electrons from an electrode, the simulated yield was significantly increased to 1 mol/mol l-lactate, which was 57% higher than the its theoretical maximum yield. This can be explained by the significantly increased fluxes of the threonine bypass (Figure 9) (Lin et al., 2015). The threonine bypass is able to not only utilize additional reducing equivalents to fix extracellular CO_2_ (4.06 mmol/gDW/h, Figure 9), but also re-assimilate formate released by PFL (4.06 mmol/gDW/h, Figure 9), and generate the key precursor acetyl-CoA with a higher yield than that obtained merely via the carbon-losing PFL pathway. As a result, 3HB can be generated at a rate of 4.06 mmol/gDW/h *in silico*, which represents the yield of 1 mol/mol l-lactate. It is worth mentioning that the 3HB yield would not be infinitely improved in MESs of MR-1, which might be owing to the lacking of key intermediate metabolite formate. Finally, these results indicated that metabolic regulation of the threonine bypass will be an important aspect of MR-1 for enhancing the yield of chemicals in future MES.

Since glycerol turns out to be the best substrate for election generation (Figure 6), we also simulated the product synthesis using glycerol in MESs (Supplementary Datasheet S4, Supplementary Table S10). Three major findings were obtained: 1) Without inward electrons, the product yields using glycerol are higher that using l-lactate, which is consistent with that in electron generation. 2) With inward electrons, the product yields using glycerol or l-lactate are equivalent, which means that extra electron supplying might eliminate the difference caused by different reducing degree in substrates. 3) The requirement of inward electrons for the same product is significantly lowered when using glycerol rather than l-lactate. This indicates that glycerol might still be a better substrate in MESs, which lowers the demand for the electron uptake rate of *S. oneidensis* strains.

## Discussion

Genome-scale metabolic modeling is a systematic biology approach for the construction of a framework for the integrative analysis of metabolic functions of a microorganism. In this study, we reconstructed a well-curated genome-scale metabolic model for the facultative dissimilatory metal-reducing bacterium *S. oneidensis* MR-1, a model EAB with applications in energy-generation and bioelectrochemical product synthesis. The model iLJ1162 comprised 1,162 genes, 1837 metabolites and 2078 reactions located throughout the extracellular, periplasm and cytosol compartments. GPRs extracted from various databases were used to extend the model, so that iLJ1162 can cover 27.8% (1,162/4196) of the annotated ORFs, which is significantly higher than the 19% of iMR1_799 (799/4196) and 18.9% of iSO783 (783/4196) (Table 3). In addition, the curation of the respiratory chain not only enables the iLJ1162 model to simulate normal cellular energy production, but can also simulate the use of a variety of terminal electron acceptors. This indicated that iLJ1162 has the potential to analyze the evolution process of respiratory versatility in *Shewanella*. The replacement of the biomass composition and gapfilling enabled iLJ1162 to specifically simulate the growth and metabolic state of *S. oneidensis* MR-1. At the same time, the calculation results of the growth rate (0.105 h^−1^), which are the closest to the experimental data (0.085 h^−1^), indicated that iLJ1162 could accurately simulate the growth and metabolism of *S. oneidensis* MR-1. Furthermore, 289 substrates that were screened based on phenotypic assays were used to elevate and validate the model. The results of iLJ1162 reached 86.9% concordance with the wet-lab results, while iMR1_799 and iSO783 reached 62.3 and 61.9% respectively. This demonstrated that iLJ1162 could more accurately simulate substrate utilization than earlier models. Throughout the model construction process, the quality and accuracy of the model was gradually enhanced, and it achieved a high score of 90 in the MEMOTE evaluation. This not only proves the high quality of iLJ1162, but also indicates the rationality and comprehensiveness of the model construction process.

**TABLE 3:** Comparison of results among three GEMs of *S. oneidensis* MR-1.

Model	Genes	Metabolites	Reactions	Coverage of ORFs (%)	Prediction accuracy ratio (%)		Growth rate (h^−1^)	MEMOTE score
					Electron acceptor spectrum (%)	Substrate spectrum		
iSO783	783	634	870	18.7	56.25	61.9%	0.108	35
iMR1_799	799	647	933	19.0	62.5	62.3%	0.190	20
iLJ1162	1,162	1,818	2094	27.8	100	86.9%	0.105	90

MFCs based on bio-electrochemical processes initially gained significant attention due to their unique potential to generate energy. In MFCs, the electron pool of EAB plays an important role in electricity production. In this work, we simulated the potential electron generation capacity of different substrates, screened genetic targets for increasing intracellular NADH regeneration, as well as the transfer pathway for electrons from NADH to enter the respiratory chain, providing guidance for controlling and optimizing the metabolic capacity and activity of MR-1 to improve the EET rate. Firstly, we analyzed the electrons produced from all the carbon sources that can be utilized by MR-1 (Figure 6). Glycerol, which is a heterologous substrate for *S. oneidensis* MR-1, was predicted to be the optimal substrate for electron generation. Moreover, the FSEOF algorithm was used to find targets for increasing the ratio of NADH/NAD^+^. When using l-lactate as electron donor, PflB, FDH and MDH were predicted as engineering targets for efficient electricity generation, which was consistent with previous experimental results (Li et al., 2018). This indicates that our simulation results are meaningful, and other targets also merit further wet-lab verification to improve the electricity production of *S. oneidensis* MR-1 in MFCs. In future, a systematic simulation based on iLJ1162 using more algorithms such as OptForce (Ranganathan et al., 2010), MOMA (Segrè et al., 2002) and FVSEOF (Park et al., 2012), is expected to provide a more comprehensive guidance for rational design of novel BESs. Notably, the serine bypass was predicted to be the main target distribution pathway regardless of whether lactate or NAG was used as the substrate. Under anaerobic conditions, succinate cannot be directly converted to fumarate, resulting in interruption of the TCA cycle and an increase of metabolic flow into the serine bypass to produce NADH. Thus, controlling the expression level of the targets in the serine bypass may enhance electron generation. Furthermore, increasing the flow of NADH to the respiratory chain could also increase the EET rate. Tao et al. (2016) found that the MFC current density produced by an engineered strain with upregulation of non-proton-pumping NADH dehydrogenase was 3.3-fold higher than that of wild-type MR-1. Our results indicated that l-proline and d-lactate could serve as electron-transfer intermediates between NADH and quinol, and the functions of NDHs could be replaced by corresponding enzymes (LdhA/Dld and ProC/PutA). The function of d-lactate was confirmed by Watanabe et al. (Nakagawa et al., 2015; Kasai et al., 2019) using glucose or NAG as carbon source. However, to the best of our knowledge, none of the studies has reported that l-proline can be used as a temporary sink for electron transfer in *S. oneidensis* MR-1. Thus, if genetic manipulation is feasible, controlling the expression levels of LdhA/Dld and/or ProC/PutA might increase the rate of EET.

MESs represents a sustainable platform that converts waste into resources, using microorganisms within an electrochemical cell (Gajda et al., 2021). As initially reported by Ross et al. (2011), Rowe et al., 2017 as well as Tefft et al. (Tefft and Teravest, 2019), the electrons from cathode are confirmed to be transferred to MQ7, from where they are further passed to NAD^+^ to form NADH with proton-motive force from a light-driven proton pump (proteorhodopsin). These works open up the possibility to develop efficient MESs based on MR-1 chassis. Here, we used the iLJ1162 to profile the optimal biosynthesis pathways for six different chemicals in MESs. Notably, these optimal pathways are quite different with those without inward electrons. Taking 3HB as an example (Figure 9), the NADH derived from inward electrons can not only save the fluxes once into the serine bypass and PP pathway, but also provide extra reducing force for CO_2_ and formate fixation through the threonine bypass. Thus, the simulated yield with inward electrons exceeded its maximum theoretical yield (Table 2). It is worth mentioning that the threonine bypass has been proven to be an efficient engineering target for improving for acetyl-CoA derived poly (3-hydroxybutyrate) production in *E. coli* in our previous work (Lin et al., 2015). Therefore, our simulation results can also promisingly guide further engineering of MR-1 chassis for efficient MESs in future.

## Conclusion

In this study, we reconstructed a high-quality metabolic model of *S. oneidensis* MR-1. The model, named iLJ1162, is more comprehensive in scope than previous models and updated based on the latest gene annotations, databases and literature. The model validation was achieved by comparing model predictions to experimentally obtained results or the literature, and demonstrated a high degree of concordance. As a genome-scale metabolic network model, iLJ1162 was used to simulate and analyze the performance MR-1 in BESs from a systems perspective. Particularly in MFCs, iLJ1162 was used to analyze the electricity production capacity of 20 substrates. The model identified more than 31 genetic modification targets and two alternative NDHs pathways, which could enhance the electricity production capacity and EET rate of MR-1 in MFCs. In addition, iLJ11621 was also successfully used to simulate and analyze the synthesis of six high-value chemicals in MESs, offering preliminary theoretical exploration to guide the feasible direction of bioelectrochemical synthesis using MR-1. This advanced and comprehensive genome-scale metabolic model offers a new platform for a better understanding the metabolic features of *S. oneidensis* MR-1 at a systems-level, enabling the development of improved strains for BESs applications.

## Data Availability

The datasets presented in this study can be found in online repositories. The names of the repository/repositories and accession number(s) can be found in the article/Supplementary Material.
