# Supplementary material for: Reconstruction of a Genome-Scale Metabolic Network for Shewanella oneidensis MR-1 and Analysis of its Metabolic Potential for Bioelectrochemical Systems
Source: Front Bioeng Biotechnol. 2022 May 12;10:913077. doi: 10.3389/fbioe.2022.913077 (PMC9133699; doi:10.3389/fbioe.2022.913077)
Supplement: Supplementary file 3 [file DataSheet1.ZIP › Supplementary file 1/Figure S1.docx]

Reconstruction of a Genome-scale Metabolic Network for *Shewanella oneidensis* MR-1 and Analysis of its Metabolic Potential for Bioelectrochemical Systems

Jiahao Luo^1, 2, †^, Qianqian Yuan^2, †^, Yufeng Mao^2^, Fan Wei^2^, Juntao Zhao^1^, Wentong Yu^1, 2^, Shutian Kong^1^, Yanmei Guo^2^, Jingyi Cai^2^, Xiaoping Liao^2^, Zhiwen Wang^1, *^, Hongwu Ma^2, *^

^1^ Key Laboratory of Systems Bioengineering (Ministry of Education); SynBio Research Platform, Collaborative Innovation Center of Chemical Science and Engineering (Tianjin); Frontier Science Center for Synthetic Biology (Ministry of Education); Department of Biochemical Engineering, School of Chemical Engineering and Technology, Tianjin University, Tianjin 300072, China.

^2^ Biodesign Center, Key Laboratory of Systems Microbial Biotechnology, Tianjin Institute of Industrial Biotechnology, Chinese Academy of Sciences, Tianjin 300308, China.

† These authors contributed equally to this work.

*** Correspondence:**Zhiwen Wang
zww@tju.edu.cn

Hongwu Ma
ma_hw@tib.cas.cn


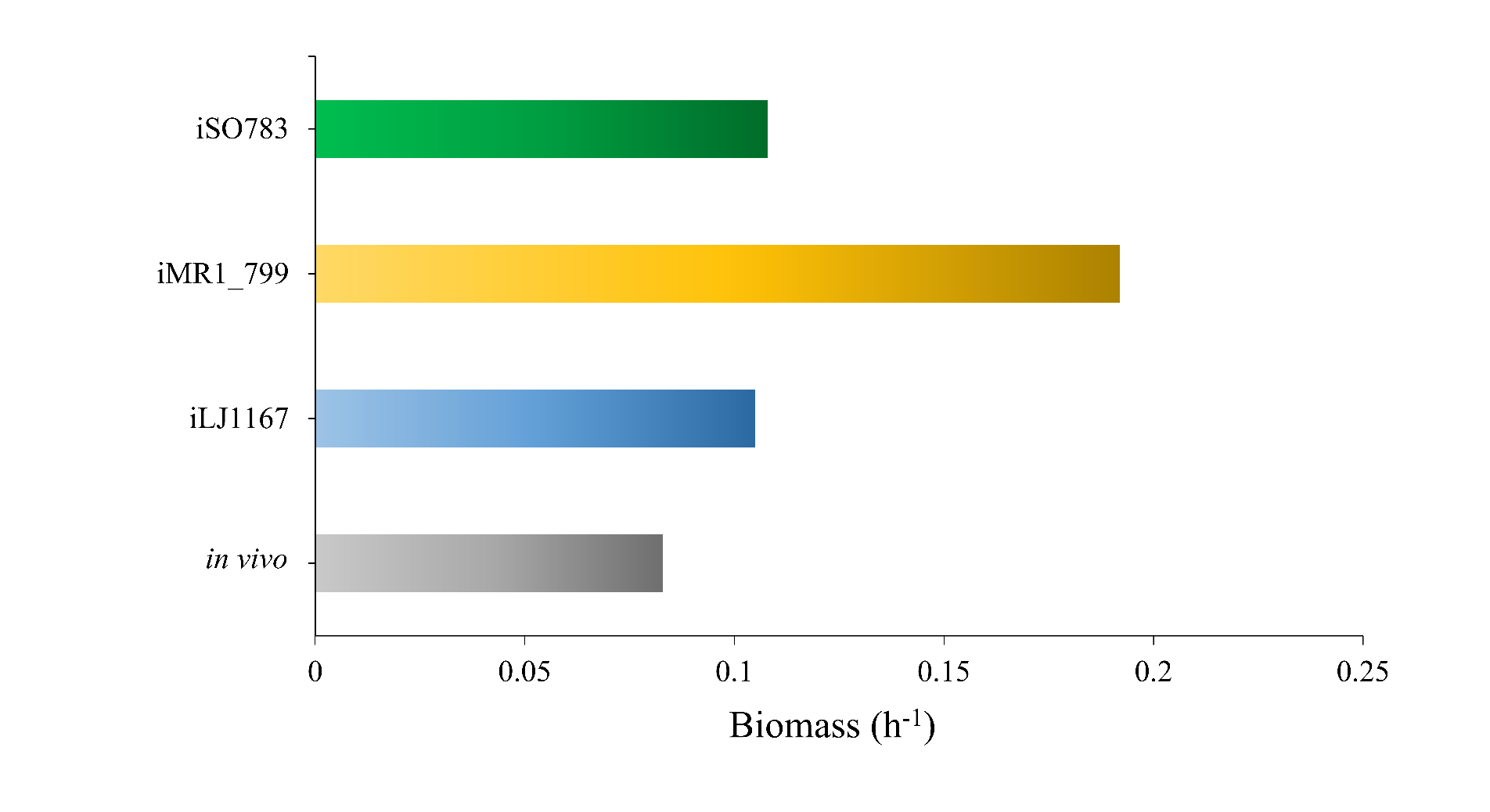


**Supplementary Figure S1. Comparison of *in vivo* and *in silico* growth rates of *S. oneidensis* MR-1.**
